# Recent advances in understanding chronic rhinosinusitis endotypes

**DOI:** 10.12688/f1000research.16222.1

**Published:** 2018-12-07

**Authors:** Eric F. Succar, Justin H. Turner

**Affiliations:** 1 Department of Otolaryngology-Head and Neck Surgery, Vanderbilt University Medical Center, Nashville, TN, 37232, USA

**Keywords:** cytokine, T-helper cell, phenotype, nasal polyps, endotype, cluster

## Abstract

Chronic rhinosinusitis (CRS) is a heterogeneous inflammatory disease with an as-yet-undefined etiology. The management of CRS has historically been phenotypically driven, and the presence or absence of nasal polyps has frequently guided diagnosis, prognosis, and treatment algorithms. Research over the last decade has begun to question the role of this distinction in disease management, and renewed attention has been placed on molecular and cellular endotyping and a more personalized approach to care. Current research exploring immunologic mechanisms, inflammatory endotypes, and molecular biomarkers has the potential to more effectively delineate distinct and clinically relevant subgroups of CRS. The focus of this review will be to discuss and summarize the endotypic characterization of CRS and the potential diagnostic and therapeutic implications of this approach to disease management.

## Background

Chronic rhinosinusitis (CRS) affects more than 4% of the US population and results in substantial economic burden to the health-care system ^1– 4^. The medical and surgical management of CRS has changed incrementally over the last two decades; however, the recent introduction of humanized monoclonal antibodies that target individual inflammatory mediators has the potential to change the course of treatment for millions of patients. Treatment success will likely depend on appropriate patient selection and improved understanding of CRS pathophysiology. Recent research has begun to explore the heterogeneity of CRS by using unbiased approaches that incorporate clinical, demographic, and inflammatory variables. These analyses have moved beyond simple phenotypic classifications of CRS and instead have set out to differentiate CRS on the basis of cytokines and other inflammatory mediators that may be more relevant to disease mechanisms. The identification of multiple putative inflammatory endotypes, with relative consistency between studies, suggests that this may be a valid approach for characterizing CRS inflammation, predicting treatment outcomes, and identifying patients who may benefit from targeted therapeutics. This review will summarize recent advances in the identification and characterization of CRS endotypes and review potential impacts on patient care.

## Chronic rhinosinusitis pathophysiology

Unfortunately, research into CRS disease mechanisms has been limited by a lack of validated animal models and the complexity of the disease itself. Although the pathophysiology of CRS is incompletely defined, current evidence suggests that both host (altered innate and adaptive immunity and genetics) and environmental (microbial pathogens and allergic sensitivity) factors likely play a cooperative role in disease initiation and progression ^5– 8^. CRS is largely a mucosal disease with locoregional inflammation in the paranasal sinuses and upper airway that does not typically extend to the systemic circulation. This conclusion is supported by studies that show a lack of specific correlation between peripheral inflammatory markers and CRS subtypes ^9– 11^. Patients with CRS frequently have altered mucosal innate immune responses and a propensity toward infection or colonization (or both) by microbial pathogens ^12, 13^. This may derive from epithelial barrier dysfunction, a common finding in CRS that potentially results in compromised host defense and reduced tolerance to common microbial antigens ^14– 16^. Epithelium-derived cytokines and chemokines subsequently can drive T cell-, B cell-, and innate lymphoid cell-mediated immune responses ^17– 20^. T helper cell responses in CRS are highly variable, and a combination of Th1, Th2, Th17, and likely other T helper subsets drives the production of specific cytokines and results in a persistent inflammatory state ^21– 23^. Furthermore, CRSwNP is frequently associated with infiltration by plasma cells and increased local production of immunoglobulin E (IgE) antibodies ^24– 26^. Differential tissue infiltration by eosinophils or neutrophils (or both) is typical in CRS and governed by T cell-mediated immune responses and the resulting cytokine milieu.

The role of eosinophilic/Th2 inflammation in the pathophysiology of CRSwNP has been the focus of significant research. Currently, monoclonal antibodies that are being investigated for use in CRS target eosinophilic/Th2 pathways ^27^. Neutrophils have been shown to drive inflammation in subsets of steroid-resistant CRSwNP and CRSsNP ^28, 29^. Understanding how neutrophils impact mucosal barriers, tissue remodeling, and polyp formation and delineating immune factors that drive the process have potential diagnostic and therapeutic implications. Recent studies suggest that interleukin-1 beta (IL-1β), IL-6, IL-8, oncostatin M, transforming growth factor-beta, and IL-36γ play a role in the pathophysiology of neutrophilic inflammation in CRS ^28, 29^. A greater understanding of these pathways may guide future investigation into the use of monoclonal antibodies targeting neutrophilic pathways in CRS.

## Chronic rhinosinusitis phenotypes

Clinical phenotypes use a combination of observable features to differentiate patients with the same diagnosis. CRS phenotyping most commonly differentiates patients on the basis of the presence or absence of nasal polyps, as this is an easily identifiable trait that can be recognized in clinical practice. However, additional phenotypes—including CRS associated with aspirin-exacerbated respiratory disease, allergic fungal rhinosinusitis, and cystic fibrosis-associated CRS—have been described and are commonly used clinically. Although these phenotypes help to distinguish patients on the basis of disease behavior and have an underlying biological basis, they generally do not adequately explain underlying disease pathophysiology. For example, CRS phenotypes have typically been associated with characteristic immune signatures, with CRSsNP typically being linked with elevation of Th1-associated cytokines and CRSwNP being associated with a Th2 or mixed Th1/Th2 response ^30, 31^. However, this rigid dichotomy is being questioned, and it is now commonly accepted that both CRSsNP and CRSwNP can present with a combination of Th1-, Th2-, and Th17-associated signatures ^21, 32– 34^. There is also substantial geographic variability to underlying inflammatory burden that cannot be explained by differences in phenotype alone ^23^.

## Chronic rhinosinusitis endotypes

It is clear that phenotypic classification of CRS does not adequately describe the complexity of the disorder ^5^ and that multiple unique inflammatory subtypes or endotypes likely exist within each phenotype ^35^. Not surprisingly, outcomes guided by phenotypic classification are often difficult to predict ^36^. The identification of inflammatory CRS endotypes has the potential to guide treatment and offer alternatives to patients who are refractory to traditional medical and surgical therapies ^37^. This approach has been used successfully in a number of inflammatory diseases, including eosinophilic esophagitis and asthma ^38– 42^. As summarized previously, targeted characterization of inflammatory markers has identified Th1-, Th2-, and Th17-driven inflammatory signatures in subsets of patients with CRS. In contrast, more recent studies have used unstructured approaches that incorporate multi-dimensional data to identify clusters of patients with presumably similar mechanisms of disease ^32, 33, 43, 44^. Whereas the number of identified clusters has varied between studies, the underlying characteristics of the clusters have resulted in generally consistent findings ( Table 1). In a multi-institutional European study, Tomassen *et al*. used hierarchical cluster analysis to analyze 14 inflammatory mediators in surgically obtained tissue from 173 patients with CRS and identified 10 distinct CRS clusters ^32^. Clustering was driven primarily by (1) eosinophilic and Th2-related markers (IL-5 and IgE), (2) neutrophilic or pro-inflammatory mediators or both (IL-1β, IL-6, IL-8, and myeloperoxidase), (3) Th17/Th22 markers (IL-17A, IL-22, and tumor necrosis factor-alpha), and (4) the Th1 cytokine interferon-gamma (IFN-γ). About 60% of patients had a Th2-high signature with a high prevalence of nasal polyposis and asthma. In a similar approach, Liao *et al*. analyzed 246 Chinese patients with CRS and incorporated both inflammatory biomarkers and clinical/demographic variables in the clustering model ^44^. Seven distinct clusters were identified; however, in their study, in contrast to the study by Tomassen *et al*., fewer than 20% of patients carried a dominant Th2 signature. The remaining clusters were characterized primarily by either mild inflammatory burden or neutrophilic inflammation. The authors also incorporated treatment outcomes into their analysis by comparing the frequency of “difficult-to-treat” CRS in each cluster. The Th2-dominant cluster was associated with severe clinical disease and poor treatment outcomes. However, a comparably larger number of patients with difficult-to-treat CRS were actually found in clusters associated with neutrophilic disease and elevated pro-inflammatory cytokines. The study also found that elevated levels of IL-10 were associated with less severe, more treatment-responsive disease ^44^.

**Table 1.** Summary of recent studies identifying chronic rhinosinusitis endotypes.

Authors	Year	Material analyzed	Sample size	Biomarkers	Clusters/endotypes
Tomassen *et al*. ^32^	2016	Tissue (sinus mucosa/polyps)	173 patients with CRS, 89 controls	14	10
Divekar *et al*. ^43^	2017	Tissue (ethmoid)	26 patients with CRS, 6 controls	41	3
Liao *et al*. ^44^	2018	Tissue (ethmoid/polyp)	246 patients with CRS, 16 controls	39	7
Turner *et al*. ^33^	2018	Mucus	90 patients with CRS, 17 controls	18	6

CRS, chronic rhinosinusitis.

CRS endotyping has the potential to impact patient care by identifying individuals with potentially recalcitrant disease and predicting treatment outcomes. A recent pilot study by Divekar *et al*. incorporated a simple, commercially available immunoassay to identify putative CRS inflammatory endotypes in an approach that ultimately could be incorporated into clinical practice ^43^. Tissue analysis of 41 different inflammatory mediators in 26 patients with CRS identified three groups characterized by Th1/Th17 cytokines (IL-17A, granulocyte-colony stimulating factor, IL-8, and IFN-γ), Th2 cytokines (IL-5, IL-9, IL-13, and eotaxin), and growth factors (platelet-derived growth factor and vascular endothelial growth factor), respectively. The comparatively smaller number of identified clusters was likely secondary to a small sample size, as the number and stability of clusters identified by unsupervised approaches are typically highly dependent on the number of both input variables and subjects. Whereas this and prior studies specifically analyzed sinonasal tissue, a recent prospective study by Turner *et al*. identified putative inflammatory endotypes using mucus alone ^33^. This minimally invasive approach could be more efficiently incorporated into clinical practice and has the potential to assign patients to disease clusters without attaining tissue and prior to any surgical intervention. The authors assessed 18 different inflammatory mediators in 90 patients with CRS and identified six disease clusters. In that report, similar to others, most patients were assigned to a Th2-dominant cluster, a pro-inflammatory cluster with a neutrophilic signature, or clusters with low overall inflammatory burden. Most importantly, the study found that clusters were predictive of postoperative improvements in disease-specific quality-of-life scores.

## Therapeutic implications

The studies outlined above suggest that identification and characterization of CRS endotypes ultimately could both improve our understanding of CRS pathophysiology and augment patient care. Although individual biomarkers have some ability to classify patients or predict outcomes, they generally lack the sensitivity and specificity required to characterize patients with such a heterogeneous inflammatory disease ^45– 49^. New classes of biotherapeutics that target individual cytokines and inflammatory pathway components are becoming increasingly available, and the ability to identify patients who may benefit from such therapies will be an essential step toward a more personalized approach to care. Several humanized monoclonal antibodies developed primarily for the treatment of severe asthma—including mepolizumab (anti-IL-5), omalizumab (anti-IgE), and dupilumab (anti-IL-4/13 receptor)—appear to also have efficacy for the treatment of nasal polyposis ^50– 53^. However, treatment response has been highly variable, suggesting a subgroup effect that may be endotype-driven. For example, a small randomized trial evaluating the efficacy of the humanized anti-IL-5 antibody reslizumab for the treatment of nasal polyposis resulted in a response in only half of patients who received treatment. However, it was noted that increased nasal IL-5 levels were highly predictive of treatment response ^54^. A similar therapeutic window has been noted for omalizumab, with efficacy in patients with CRS being moderately dependent on peripheral IgE levels ^50, 55^. Characterization of CRS endotypes has also highlighted the need for novel and non-Th2-directed therapeutic interventions, particularly given that a minority of patients carry a strongly Th2-dominant immune signature. Pro-inflammatory neutrophilic inflammation identified in a large subset of patients may also represent a recalcitrant CRS subpopulation that could benefit from targeted therapies ^32, 33, 44^. Based on this characteristic inflammatory signature, these patients would also be expected to be less responsive to topical or systemic corticosteroids, further highlighting the need for novel interventions in this group. It is further likely that the identification of uncharacterized CRS endotypes or unique immune signatures will lead to the recognition of novel mechanisms of disease and additional targeted therapies going forward.

## Conclusions

Recent identification and characterization of inflammatory CRS endotypes have improved our understanding of CRS pathophysiology and highlighted an area in need of further investigation. Future studies will need to determine whether identified disease clusters truly represent unique endotypes or whether patients present along a continuum with features consistent with multiple endotypes or inflammatory pathways. The stability of disease clusters and endotypes over time will also need to be confirmed, as will their association with medical or surgical treatment. The role of CRS endotyping in clinical practice has yet to be defined but likely has the potential to identify clinically relevant subgroups, guide personalized therapeutic algorithms, and predict outcomes.
